# Optimization of Residential Landscape Design and Supply Chain System Using Intelligent Fuzzy Cognitive Map and Genetic Algorithm

**DOI:** 10.1155/2022/6321101

**Published:** 2022-09-13

**Authors:** Tingyin Deng

**Affiliations:** Sichuan University of Science & Engineering, Zigong, Sichuan 643000, China

## Abstract

This work intends to optimize residential landscape design and Supply Chain (SC) network systems. First, Fuzzy Cognitive Map (FCM) intelligent assistance and genetic algorithm (GA) are used to study residential landscape design and its integration with SC deeply. Weight matrix interactions are employed to implement iterative inference for FCM. The functions are transformed to unify variables of different scopes. Subsequently, a weighting method is proposed to deal with the disadvantage of the simple average method being too general. In addition, the Hebbian learning algorithm is used to adjust the state nodes and the connection weights. Finally, according to the fitness function of the GA and logistic regression (LR) model, residential landscape design and SC are combined. The simulation experiment results show that the causal relationship analysis between SC networks under fuzzy cognition shows that the state errors of each specific situation are 0.21, 0.16, and 0.24, respectively. The total average error is 0.21 in the case of multiple iterations. The average error of the result vector under fuzzy cognition and the operation of the actual result is 0.20, 0.15, and 0.24, respectively, and the error value is much reduced. The simulation accuracy of the GA-LR method for residential landscape design is improved from 77% to 84.7%. The “kappa coefficient” is also improved to 82.3%. The conclusion shows that the weight matrix is used to analyze the high-quality performance of landscape design according to the specific situation of SC. For each specific case, FCM is effective in reducing errors over multiple iterations. Under the GA-LR method, fewer geographic location types and larger accuracy deviations can improve the simulation accuracy.

## 1. Introduction

As human needs evolve, techniques and styles change, and so do landscape design trends. Landscape architecture in the future will not only be limited to landscape design, but will also provide a series of solutions around social, economic, environmental, and other issues. Technological innovation and sustainable development means can improve people's quality of life. The technology used in the smart landscape is mainly modern information technology, but also digital technology. The digital landscape is the main body of the smart landscape. The application of digital technology and methods can break through the limitations of traditional design techniques and construction materials and greatly release the creativity of landscape planners. Many scholars believe that Supply Chain (SC) [1–3] is a process often used by enterprises. The actual process is that the raw materials are processed by the enterprise and finally transferred to the customers through various channels such as sales and various forms of business activities. However, with the rapid development of science and technology and the rapid progress of society, SC has long been not only used in business. At present, the SC functional network structure model is very helpful for residential landscape design [4–6]. Compared with developed countries in Europe and America, the development of residential landscape design in China is still at a low level [7–9]. For further development, China needs to make up the gap with foreign residential landscape design standards [10–12] and optimize the practical application of SC systems in related industries.

International scholars have important research theories on optimizing residential landscape design and SC systems [13–15]. There are more than 10,000 articles about landscape design in the Chinese master thesis. These research directions are standardized residential landscape design. In addition, contemporary real estate enterprises are investigated on the spot, and some historical and common problems in residential landscape design are further summarized. Chinese residential landscape design is too simple and lacks humanistic considerations. It is more inclined to residential attributes and pays less attention to the spatial, humanistic, and temporal value of landscape facilities. Foreign scholars have conducted special studies on the quality of living space [16–18] and European standards. They studied landscape design, planted seedlings, and developed norms and standards for ornamental plants. In addition, related scholars have also specialized in research strategies for 3D landscape representation. They perform 3D modeling [19–21] to seek a standardized study of landscape design. In water-constrained countries, waterwise landscaping is considered an important landscaping method to conserve water. However, there is still a concern and a lack of knowledge about people's preferences and the factors that influence them.

Nazemi Rafi et al. [22] examined the effect of landscape factors of plant combination (level 6) and cover type (level 3) on the preferences of 207 respondents. After obtaining the preferences that participants assigned to each plot, they used regression analysis to determine the effect of landscape attributes on overall preferences. Results showed that flowering plants were more popular (*p* ≤ 0.01) and less expensive (*p* ≤ 0.01) in water landscapes containing only herbaceous plants compared to other designed landscapes. The Visible Green Index (VGI) reflects the degree of greening from human vision to affect health and well-being and has gradually become a new type of urban green space index. Zhu et al. [23] designed and conducted an experiment to evaluate human responses to scenes from panoramic pictures of residential green spaces projected by virtual reality. This study provides a certain theoretical basis for the landscape planning of residential areas, which is helpful to improve the VGI theory and promote its establishment as a new type of urban green space index. O'Neill and Maravelias [24] mentioned in *Towards Integrated Landscape Design and Biofuel Supply Chain Optimization* the integration of landscape design with SC networks [25–27] to determine the synergy of the systems. The research of relevant international scholars makes it possible to optimize the residential landscape design and SC system.

In the research results of relevant scholars in this field, although the theory of the combination of residential landscape standardization design and SC system network is proposed, the research direction is also standardized residential landscape design. These theories do not involve optimizing residential landscape design and SC systems. Therefore, this study innovatively uses the intelligent assistance of a Fuzzy Cognitive Map (FCM) and genetic algorithm (GA) further to optimize the residential landscape design and SC system. The innovation lies in the optimization of the SC system, a new design for the residential landscape.

## 2. Methods

### 2.1. FCM

**Figure 1:**
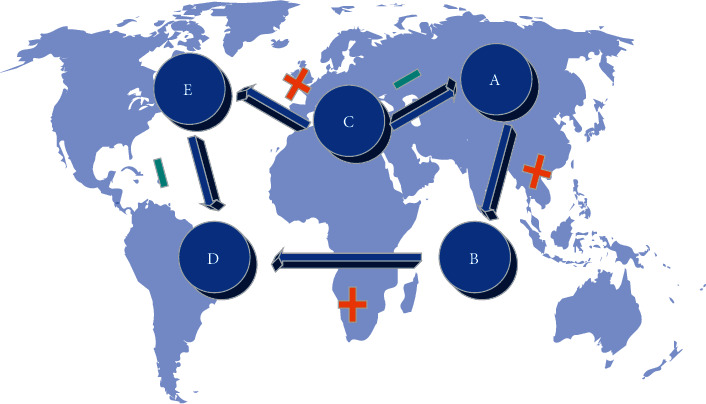
Simple FCM.

The function of FCM is to solve complex problems. Uncertain decision-making can impact decision-makers, and FCM constructs an important process of people's decision-making and analyzes its construction patterns to explain the external world. Cognitive maps have a variety of ways to build high-quality and often accurate results. The general principle of FCM is that the path of adjacent connection points in the cognitive graph is a vector arc segment, which expresses the causal relationship between nodes [28–30]. If any two connection points in the cognitive graph extend through a directed path, and the sign between the connection points is positive, it means that the relationship between them is positive. Otherwise, their relationship is an inverse relationship.  Figure 1 shows a simple FCM.

In Figure 1, a fuzzy cognitive graph with *n* nodes, each node represents a concept in the system. This concept can be events, goals, trends of the system, etc. Each concept characterizes its properties using state values. Directed arcs represent the causal influence relationships between concepts. The weight *w*_*ij*_ reflects the degree of causal influence. *w*_*ij*_>0 means that the increase in *C*_*i*_ will lead to the increase of *Cj*. There is a positive causal relationship between *C*_*i*_ and *C*_*j*_, which is indicated by “+” in Figure 1; *w*_*ij*_<0 means that the increase of *C*_*i*_ will increase *C*_*j*_ decreases. There is a negative causal relationship between *C*_*i*_ and *C*_*j*_, that is, the part indicated by “−“ in Figure 1; *w*_*ij*_ = 0 means that there is no causal relationship between *C*_*i*_ and *C*_*j*_. *C*_*i*_ and *C*_*j*_ are represented by *A*, *B*, *C*, *D*, and *E* in Figure 1.

Based on the scenario studied in the present work, the discrete-time Markov decision process with continuous action space can be used to represent the optimal stochastic control problems of residential landscape allocation and location factor scheduling. Because of the complex transformation of the external environment, the residential landscape module cannot obtain accurate transformation information. Besides, the optimal solutions with low complexity are difficult to obtain by the general algorithm or equation through the previous methods. Therefore, the resource fuzzification of FCM under the reinforced deep learning (DL) algorithm is studied below to optimize the residential landscape design further. In the case of discrete time, the optimization aims at the conventional reinforcement learning problem composed of a residential landscape design environment and intelligent fuzzy cognitive assistance. The real-time reward is based on the timely action taken by the intelligent module after receiving the observation results in each process. The state space is the product of the resource allocation scenario of residential landscape design, that is, the current environmental state of the intelligent module. According to the DL model of the large-scale residential landscape, in equation (1), *cx* represents the data transmission direction and pointer; *w*_*ij*_ represents the weight; *x*_*i*_(*t*) represents the object's value at the current moment; *x*_*i*_(*t*+1) represents the value of the object at the next moment. The derivation process of the cognitive graph is not complicated, and its key function is to grasp the concept nodes and weight matrix. Equation (1) represents a specific iterative function.

(1) $$\begin{array}{c} x_{i}\left(t+1\right)=f\left[\left(\overset{n}{\underset{\begin{array}{c} j=1\\ j\neq i \end{array}}{\sum }}w_{ij}x_{j}\left(t\right)+cx_{i}\left(t\right)\right)\right] \end{array}$$

The transformation function [31] is used to change the data useless to the system. The variation range of the transformation function is [0,1]. Equation (2) is a threshold function; equation (3) is a hyperbolic tangent function; equation (4) is a sigmoid function [32–34]; Equation (5) is a saturation function. In equations (2)–(5), *x* represents a real number between 0 and 1.

(2) $$\begin{array}{c} f\left(x\right)=\left\{\begin{array}{c} 1,x\geq 0\\ 0,x<0 \end{array}\right. \end{array}$$

(3) $$\begin{array}{c} f\left(x\right)=\tanh\left(x\right) \end{array}$$

(4) $$\begin{array}{c} f\left(x\right)=\frac{1}{1+e^{-x}} \end{array}$$

(5) $$\begin{array}{c} f\left(x\right)=\left\{\begin{array}{cc} 1\text{,} & x\geq \frac{1}{k}\\ kx\text{,} & -\frac{1}{k}\leq x\leq \frac{1}{k}\\ -1\text{,} & x<-\frac{1}{k} \end{array}\right.\text{.} \end{array}$$

Based on the general problem of simple average, the weight method is proposed, mainly used to conduct the weighted average on the simple average. Equation (6) demonstrates the principle of the method.

(6) $$\begin{array}{c} W=f\left(\overset{n}{\underset{k=1}{\sum }}b_{k}W_{k}\right) \end{array}$$

In equation (6), *W* means the final weight; *n* denotes the number of experts; *W*_*k*_ refers to the weight matrix according to the *kth* expert; *f* stands for conversion function, which is used to change all data in the weight matrix to the range between −1 and 1; *b*_*k*_ means the accuracy of matching expert experience and knowledge.

Hebbian learning algorithm. The two most important factors in Hebbian learning of FCM are the dynamic characteristics of process and environment. The value of connection weight can be further adjusted by adjusting the state node. *Wij* denotes the weight between node *cj* and node *ci*, when *xj* represents the state of node *cj* and *xi* represents the state of *ci*. Therefore, when *xj* and *xi* change, the adjustment function is shown in:

(7) $$\begin{array}{c} \Delta w_{ji}=\gamma w_{ji}^{k-1}+\eta A_{i}^{k-1}\left(A_{j}^{k-1}-sgn\left(w_{ji}^{k-1}\right)w_{ji}^{k-1}A_{i}^{k-1}\right) \end{array}$$

In equation (7), *η* means learning factor; *γ*points to the attenuation factor.

GA algorithm is used to select the values with good adaptability and pass the values with high-quality characteristics to the next generation. In equation (8), *M* stands for the population size; *F*_*i*_ accords to the fitness of monomer *i*. Equation (8) illustrates the calculation of the probability that *i* is selected.

(8) $$\begin{array}{c} P_{i}=\frac{F_{i}}{\overset{M}{\underset{i=1}{\sum }}F_{i}}\;\left(i=1,2,\ldots ,M\right)\text{.} \end{array}$$

The minimum deviation *e* means that the fitness of GA has been reduced to the minimum state. Then, its termination condition is expressed as:

(9) $$\begin{array}{c} \left|f_{\max}-f^{*}\right|<e \end{array}$$

In equation (9), *f*^*∗*^ represents the fitness goal and *f*_max_ denotes the maximum fitness. The fitness function is set to the inverse of the prediction error to train the FCM. Equation (10) manifests its expression.

(10) $$\begin{array}{c} f=\frac{1}{e}=\frac{1}{\sqrt{\left(1/m\right)\overset{m}{\underset{i=1}{\sum }}{\left(s_{i}-{\hat{s}}_{i}\right)}^{2}}}\text{.} \end{array}$$

In equation (10), *s*_*i*_designates the actual value and ${\hat{s}}_{i}$ stands for the predictive value of FCM.

(11) $$\begin{array}{c} Probability\left(gene\;j\;is\;selected\right)=\frac{f_{j}}{\overset{m}{\underset{k=1}{\sum }}f_{k}}\text{.} \end{array}$$

In equation (11), *f*_*j*_refers to the fitness value of the *j*th gene.

(12) $$\begin{array}{c} \text{minG}\left(Q,R\right)=\frac{k\lambda }{Q}+h\left(\frac{Q}{2}+R-\lambda \tau \right)+\frac{Pn\left(R\right)\lambda }{Q} \end{array}$$

In equation (12), *n(R)* means the prediction of landscape design land; *Q* accords to the amount of landscape land occupation; *R* stands for the current land stock; and *H* represents the market interest rate.

(13) $$\begin{array}{c} g\left(s_{i}^{t}\right)=\left\{\begin{array}{c} 0,ifs_{i}^{t}<a_{i}\\ \left(s_{i}^{t}-a_{i}\right)/\left\{2\left(m_{i}-a_{i}\right)\right.,ifa_{i}\leq s_{i}^{t}\leq m_{i}\\ 0.5+\left(s_{i}^{t}-m_{i}\right)/\left\{2\left(b_{i}-m_{i}\right)\right.,ifm_{i}\leq s_{i}^{t}\leq b_{i}\\ 1,ifs_{i}^{t}\geq b_{i} \end{array}\right.\text{.} \end{array}$$

In equation (13), the state value of fuzzy cognition is generally set as a real number between 0 and 1. The purpose of introducing a nonlinear function approximator into the algorithm is to better deal with the problems of continuous space and multi-dimensional state space. The ordinary linear function approximators cannot properly deal with the problems of the frequency resources of the unauthorized frequency band. In the following algorithm, the residential landscape design is the core, the SC is the main structure of the algorithm, and the supply network inputs the state into St and outputs the result. Landscape design is triggered by the subsequent trigger action of the intelligent module under the action of the surrounding environment of the residence and feeds back to the new state. Therefore, it is necessary to standardize the numbers in this range. The standardized method is the historical minimum, maximum, and average values obtained in time *t*. In equation (13), *s*_*i*_^*t*^ means the *i*th state value at time *t*.

(14) $$\begin{array}{c} a_{i}=\min_{t\in T}\left\{s_{i}^{t}\right\}\\ b_{i}=\max_{t\in T}\left\{s_{i}^{t}\right\}\\ m_{i}=\underset{t\in T}{average}\left\{s_{i}^{t}\right\} \end{array}$$

The state vector can be obtained based on the operation of fuzzy cognition [35–37], which can be expressed as an equation:

(15) $$\begin{array}{c} S_{t}=f\left(S_{t-1}\times E\right),t=1,2,\ldots \text{.} \end{array}$$

In equation (15), *f*(*S*_*t*_) refers to a transfer function whose specific purpose is to set the state values between 0 and 1; *s*_*i*_^*t*^ represents the *i*th state value at time *t*; ${\hat{s}}_{i}^{t}$ accords to the results of the fuzzy cognitive calculation. Then, the difference between ${\hat{s}}_{i}^{t}$ and*s*_*i*_^*t*^ is the error in the prediction. Furthermore, equation (16) displays the square root of all state values.

(16) $$\begin{array}{c} e_{t}=\sqrt{\frac{1}{n}\overset{n}{\underset{i=1}{\sum }}{\left(s_{i}^{t}-{\hat{s}}_{i}^{t}\right)}^{2}}\text{.} \end{array}$$

Equation (17) discloses the expression of the total prediction error of the GA.

(17) $$\begin{array}{c} \min\underset{t\in T}{\sum }e_{t} \end{array}$$

(18) $$\begin{array}{c} Probability\left(gene\;j\;is\;selected\right)=\frac{f_{j}}{\overset{m}{\underset{k=1}{\sum }}f_{k}}\text{.} \end{array}$$

In equation (18), *f*_*j*_ represents the fitness value of the *j*th weight. In the Euclidean distance, prediction results of ${\hat{S}}_{G}^{C}$ are closest to the actual target vector *S*^*T*^. The calculation is shown in the equation:

(19) $$\begin{array}{c} {\hat{S}}_{G}^{C}=arg\;\min\;\left\{distance\left(S^{T},{\hat{S}}^{T}\right)\right.\text{.} \end{array}$$

The emergence of the definition of ${\hat{S}}_{H}^{C}$ means that the analysis stops because the data collected at this time are not the category of historical data anymore.

(20) $$\begin{array}{c} {\hat{S}}_{H}^{C}=arg\;\min\;\left\{distance\left(S,{\hat{S}}_{G}^{C}\right)\right. \end{array}$$

### 2.2. Genetic Algorithm

GA is an optimization algorithm with a high utilization rate in the academic field, based on Darwin's *Theory of Evolution* and related elements in genetics. It can optimize the target. Its composition is generally divided into four aspects: maximum number of iterations, crossover rate, control parameters, fitness function, and chromosome coding. The foundation of GA is based on mathematical theorems. GA has great advantages for individuals with good fitness. The definition of a genetic algorithm is that genes and chromosomes are elements. Genes are the smallest elements that assemble into chromosomes. The form that exists on chromosomes is string encoding. Characters in a string represent genes. Its algorithmic meaning is the relationship between variable solution set and variable solution. Meanwhile, these solutions are combined to form the population of GA. LR method is a common learning algorithm whose advantage is that it has characteristics to describe nonlinear problems. Simultaneously, the LR model can usually solve common problems, so it has wide practicability. Equation (21) indicates the expression form of the sigmoid function of LR:

(21) $$\begin{array}{c} f\left(t\right)=\frac{1}{1+e^{-t}}\text{.} \end{array}$$

In equation (23), *t* is a variable and represents a linear function. Equation (22) refers to the expression of fitness function *Fit(f(x))*, which is implemented to find the minimum value of objective function *f(x)*:

(22) $$\begin{array}{c} Fit\left(f\left(x\right)\right)=\left\{\begin{array}{cc} c_{\max}-f\left(x\right)\text{,} & f\left(x\right)<c_{\max}\\ o\text{,} & else \end{array}\right.\text{.} \end{array}$$

Equation (21) denotes the expression of fitness function *f(x)*, which is adopted to find the maximum value of objective function *f(x).*

(23) $$\begin{array}{c} Fit\left(f\left(x\right)\right)=\left\{\begin{array}{cc} f\left(x\right)-c_{\min}\text{,} & f\left(x\right)>c_{\min}\\ o\text{,} & else \end{array}\right.\text{.} \end{array}$$

In equations (22) and (23), *C*_max_ and *C*_min_ mean the maximum and minimum values of the objective function, respectively. The fitness function in the present work can effectively identify all chromosomes and find unsolved and normal chromosomes. If the identification degree between them is not high, the value of the fitness function should be adjusted to ensure normal identification. This is a very important step in the design of fitness functions.

Simple genetic algorithm (SGA) has three operators: crossover operator, selection operator, and mutation operator. The selection operator refers to the operator that can simulate biological evolution, which can decide whether to retain the individual according to the value of the fitness function. Generally speaking, the probability of being selected is closely related to fitness. The higher the fitness, the greater the probability of being selected. Equation (24) signifies the specific expression of the selection operator of SGA:

(24) $$\begin{array}{c} p_{i}=\frac{f_{i}}{\sum_{i=1}^{n}f_{i}}\text{.} \end{array}$$

In equation (24), *Pi* accords to the probability that monomer *i* is selected, and *fi* represents the fitness of monomer I:

(25) $$\begin{array}{c} Fit=\frac{p_{1}+p_{2}}{p_{all}} \end{array}$$

Equation (25) refers to the function of fitness *Fit*, where *P1* and *P2* represent the number of records of landscape land and non-landscape land. *Pall* stands for the total number of records in the data set of the sample. Equations (26) and (27) calculate adaptive variation rate *pm* and crossover rate *pc*, respectively.

(26) $$\begin{array}{c} p_{c}=pc_{1}-\left(pc_{2}-pc_{1}\right)*\frac{popsize-n}{popsize}\text{.} \end{array}$$

(27) $$\begin{array}{c} p_{m}=pm_{1}+\left(pm_{2}-pm_{1}\right)*\frac{popsize-n}{popsize} \end{array}$$

In equations (26) and (27), *pc1* and *pc2* point to the maximum and minimum cross rate, respectively. Parameter *n* represents the number of individuals. The change of *n* and *pc* is accompanied by the iterative process of the algorithm. The change of *pm* and *pc* is determined according to the change of *n*.

**Figure 2:**
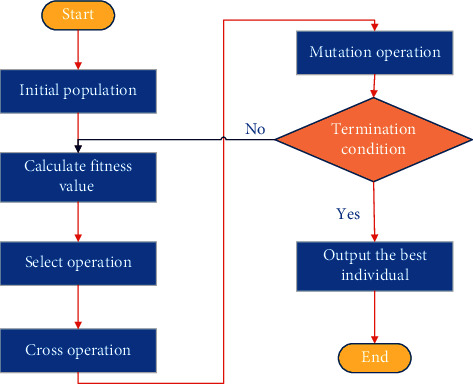
Flow of GA.

SGA [38–40] is the model's foundation in the present work. Its solution process is: first, the population is initialized, which is to determine the control parameters of GA and generate results. Second, the fitness value of the current monomer is calculated, the monomer is screened, and cross-operation is carried out. Third, the mutation operation is carried out and the iteration is judged immediately. Fourth, the optimal solution is output.  Figure 2 displays the specific flow of GA.

The disadvantage of the rule-based method is that it is often difficult for people to formulate a good rule base in an unfamiliar scene. After the membership function is established, the corresponding processing rule base is indispensable. Finally, the proposed method is simulated, and GA is used for residential landscape design and SC system optimization. The experiment uses SYNTHIA-Dataset. This dataset is a large-scale dataset of photorealistic renderings of virtual cities with semantic segmentation information, which is proposed for scene understanding in research fields such as autonomous driving or urban scene planning and garden design. The dataset provides fine-grained pixel-level annotations for 11 categories of objects (empty, sky, buildings, roads, sidewalks, fences, vegetation, poles, cars, signal signs, pedestrians, and cyclists). Thirteen thousand four hundred seven training images were extracted from the video stream. This dataset is also known for its variability, including scenes (towns, cities, highways, etc.), objects, seasons, weather, etc. Experiments on this dataset can clearly show the improvement in the performance of the residential landscape design system after the optimization of the GA.

The software and hardware environment adopted in the experiment is Matlab. The hardware and software environment settings are on one PC. The operating system is Windows 10 version. The CPU is Intel(R) Core (TM) i9-9900K CPU @ 3.60 GHz, and the memory is 8G. The programming language adopts PyThon 3.0.

## 3. Results

### 3.1. Fuzzy Cognitive Analysis of Residential Landscape SC

**Figure 3:**
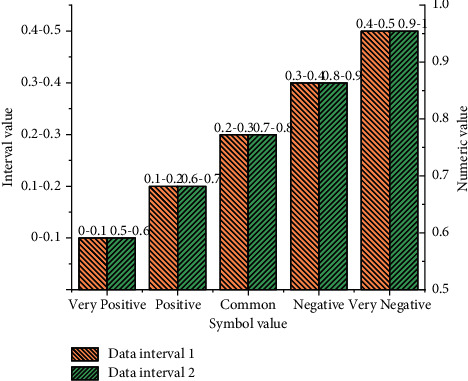
Values of key points at the fuzzy state.

Causality between SC networks under fuzzy cognition is analyzed. The storage status of landscape design land is assumed to be 1, which indicates that the actual value is much higher than that in general. In Figure 3, the interval values are the smallest when symbol values are between [0–0.1] and [0.5–0.6]. On the contrary, the interval values are the largest when symbol values are between [0.4–0.5] and [0.9–0.1]. Besides, according to the histogram, all the interval values show an increasing trend. Figure 3 shows the values of key points at the fuzzy state.

**Figure 4:**
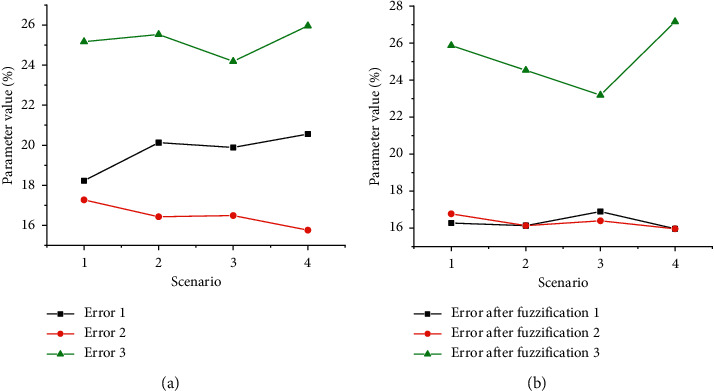
Simulation analysis of fuzzy cognition. (a) Error diagram; (b) error diagram after fuzzification.

Based on simulation experiments, the weight matrix performs well in analyzing the landscape design according to the SC.  Figure 4 demonstrates the specific situation of the simulation. For each specific case, the premise that the FCP can reach a fixed value is to carry out multiple iterations.  Figure 4(a) depicts the state error in specific cases. The error is 0.21, 0.16, and 0.24 in turn, and the total average error is 0.21. According to the result vector under FCP and the operation of actual results, the error, in this case, is reduced a lot. According to the blurred result, the average error is 0.20, 0.15, and 0.24, in turn, according to Figure 4(b). Meanwhile, compared with the initial average error, the error value is reduced to 0.19.  Figure 4 shows the fuzzy cognitive simulation analysis based on landscape design.

### 3.2. SC and GA Analysis

**Figure 5:**
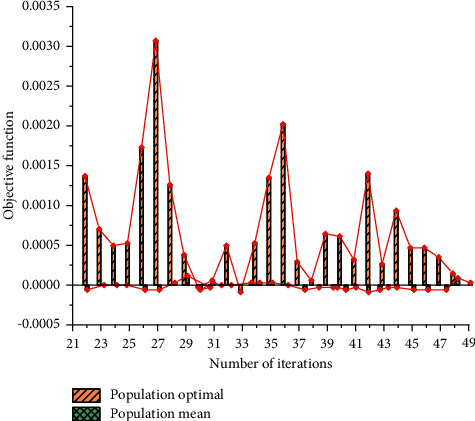
Convergence curve of GA.

GA can achieve convergence based on multiple iterations, and the mean value of the objective function can be infinitely close to zero. Therefore, samples with prediction errors close to zero can be obtained through the optimal weight combination, which is the best regression coefficient of LR.  Figure 5 shows the convergence curve of GA.

**Figure 6:**
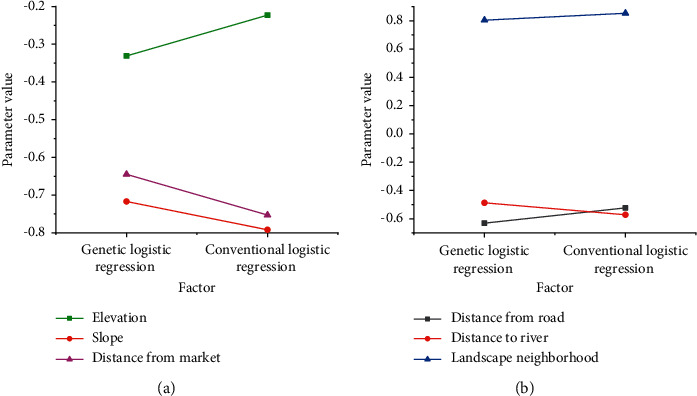
Changes of LR coefficient. (a) Genetic logistic regression coefficient. (b) Conventional logistic regression coefficient.

LR coefficients are obtained by genetic algorithm training. LR training can obtain specific regression coefficients.  Figure 6(a) and 6(b) depicts the curves of the regression coefficients under different factors, respectively. The value of the elevation factor changes from −0.33 to −0. Twenty-two under two methods, while the other two data groups in Figure 6(a) change from −0.63 to −0.74 and from −0.71 to −0.79. In Figure 6(b), the value of the factor between the landscape design and the river changes from −0.43 to −0.45, and the other two groups of data have an upward trend. Only the elevation factor plays a positive role in landscape design.

**Figure 7:**
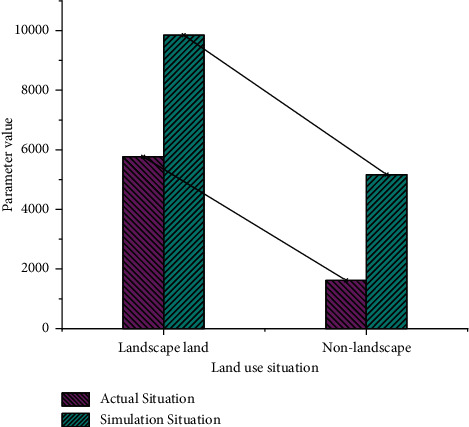
Confusion matrix for genetic LR.

**Figure 8:**
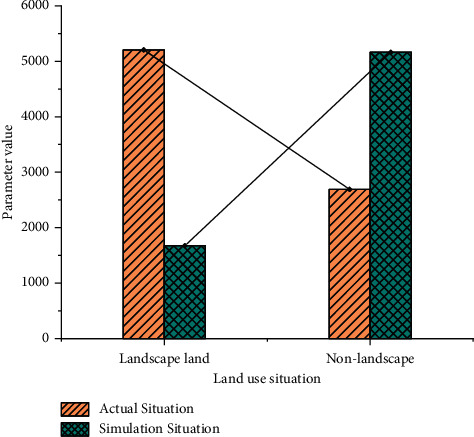
Confusion matrix for conventional LR.

Figures 7 and 8 depict the simulation results of the confusion matrix for the GA-based logistic regression (LR) and the traditional LR method, respectively. The matrix is used to calculate the simulation accuracy and Kappa coefficient.

**Figure 9:**
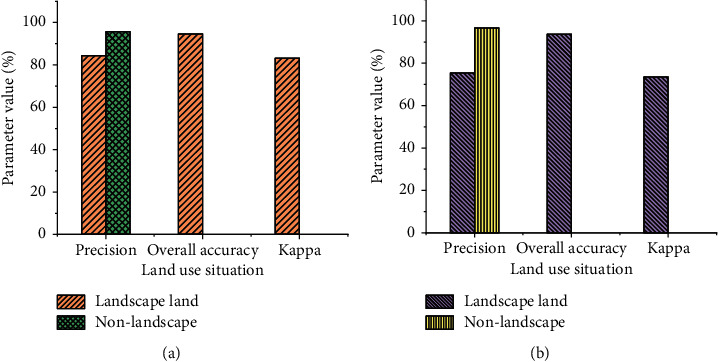
Comparison of simulation accuracy. (a) Genetic LR method; (b) conventional LR method.

In Figure 7, the actual values of landscape and non-landscape land are 5768 and 1621, respectively, and 9845 and 5169 under simulation conditions. In Figure 8, the actual values of landscape and non-landscape land are 5208 and 2691, respectively, and they are 1675 and 5163 under simulated conditions. In the research of residential landscape design, problems such as many geographical locations and insufficient sample richness should be considered to eliminate precision bias. In Figure 9, the comparison between the GA-optimized LR and the traditional LR method shows that the simulation accuracy of the proposed GA-based LR method in residential landscape design is significantly improved from 77% to 84.7%. The “Kappa coefficient” also increases to 82.3%. The proposed algorithm has better performance.

## 4. Discussion on the Application of Fuzzy Cognition and GA in Landscape Design

In Sections 3.1 and 3.2, the FCM and GA analysis results of residential landscape SC show that, through simulation experiments, the weight matrix performs well in analyzing the landscape design in the SC case. Bakhtavar et al. [41] pointed out that fuzzy cognitive graphs are widely used to analyze complex, causal-based systems in modeling, decision-making, analysis, prediction, classification, etc. Managers cannot decide to implement and allocate resources to improvement projects based on the outputs of the risk management process. Rezaee et al. [42] attempted to accurately identify and prioritize potential failures in a production process with the help of crossover using an approach based on a multi-stage fuzzy cognitive graph approach and process failure mode and impact analysis techniques. Ladeira et al. [43] aimed to develop an FCM to identify and analyze the determinants of digital entrepreneurship. Iqbal et al. [44] proposed a new GA-based feature reduction technique. This hybrid approach reduced the feature set size to 42% without compromising accuracy.

The premise of a fuzzy graph reaching a fixed value is multiple iterations. In addition, the state error of the specific case is given. This error is relatively large, and the total average error is also relatively large. The operation error between the result vector under FCM and the actual result is much reduced. Furthermore, the error after blurring is reduced compared to the initial average error. GA plays a role in improving the accuracy of optimizing the eigenvalues.

In addition, the analysis of residential landscape SC in the GA direction can achieve convergence based on multiple iterations. The mean of the objective function can be infinitely close to zero. Therefore, the optimal weight combination obtains a sample with a prediction error close to zero, that is, the optimal regression coefficient of LR. The LR coefficients obtained by GA training and the conventional LR training obtained by the maximum likelihood estimation algorithm are used to obtain specific regression coefficients. The analysis shows that the regression coefficient values obtained by the two different methods are quite different. The regression coefficient values obtained by the two methods are quite different. The results show that only the elevation factor plays a positive role in residential landscape design. Comparing GA and conventional LR methods, the simulation accuracy of the GA-LR method in residential landscape design is significantly improved. Therefore, simulated cognitive maps and GA play an irreplaceable role in residential landscape design and SC optimization.

## 5. Conclusion

The problem of combining residential landscape design with SC management and the current residential design in China is further optimized and improved to narrow the gap with foreign high-quality residential landscape design. The research work is to model, simulate and optimize residential landscape design. First, the previous research is the basis for the theoretical support. Classification and summarization are made according to the residential landscape design and the regional characteristics of geographical location, and LR is adopted as the model, which is combined with GA and the conventional LR method. Afterward, the fitting degree of this model is not enough, and the fitting ability is still weak due to the deviation of regional data and the influence of geographical location characteristics. Based on this situation, the GA-LR model is used to analyze residential landscape design and further study it. Finally, the model eventually has a good fitting effect, and its reliability is confirmed by verification and analysis. Besides, based on the SC management analysis of residential landscape design and the simulation research of SC network oriented to FCM, the logical relationship of SC network is very strong, which can well analyze the causal relationship between various nodes. Simultaneously, the complex state nodes can also be handled well further to ensure the accuracy and universality of each link. The following conclusions are drawn: (1) the weight matrix can analyze the high-quality performance of landscape design according to the situation of SC; (2) for each specific case, in the case of multiple iterations, the total average error of model simulation is small; (3) this method is very helpful to study residential landscape design; and (4) under the genetic LR method, the simulation accuracy can be significantly improved in the face of fewer types of geographical location and large accuracy deviation. The simulation data can show that the FCM constructed in the present work and the combination with the SC network system can better analyze and optimize the residential landscape design. There are still some deficiencies in the simulation accuracy calculation under genetic and conventional LR methods. A few types are considered for the geographical location of residential landscape design, and the sample richness is not enough so the accuracy deviation may be large. This problem will be the focus of follow-up research.

## Data Availability

The raw data supporting the conclusions of this article will be made available by the authors, without undue reservation.
